# Prevalence and Associated Factors of Perinatal Asphyxia in Neonates Admitted to Ayder Comprehensive Specialized Hospital, Northern Ethiopia: A Cross-Sectional Study

**DOI:** 10.1155/2020/4367248

**Published:** 2020-02-14

**Authors:** Gebrehiwot Teklehaimanot Gebregziabher, Fikaden Berhe Hadgu, Haftom Temesgen Abebe

**Affiliations:** ^1^Department of Pediatrics and Child Health, College of Health Sciences, Aksum University, Tigray, Ethiopia; ^2^Department of Pediatrics and Child Health, College of Health Sciences, Mekelle University, Tigray, Ethiopia; ^3^Department of Biostatistics, School of Public Health, College of Health Sciences, Mekelle University, Tigray, Ethiopia

## Abstract

**Background:**

Perinatal asphyxia is defined as the inability of the newborn to initiate and sustain enough respiration after delivery and is characterized by a marked impairment of gas exchange. It is one of the most common causes of neonatal mortality and morbidity. There are very few studies on perinatal asphyxia in Tigray, and so this study is aimed at assessing the prevalence and associated factors of perinatal asphyxia in Ayder Comprehensive Specialized Hospital NICU, Tigray, Ethiopia.

**Methods:**

An institution-based cross-sectional study design was conducted among neonates admitted to Ayder Comprehensive Specialized Hospital from January 1, 2016, to December 30, 2017. Medical records of 267 neonates admitted to the neonatal intensive care unit were selected by a systematic sampling method, and relevant information was collected using a checklist. The data was analyzed using SPSS version 20. Descriptive statistics were computed to determine the prevalence of birth asphyxia and sociodemographic and obstetrics data. Binary logistic regression was used to test associations between the associated factors and perinatal asphyxia. First bivariate analysis was performed to assess the association without controlling the effect of other independent variables. Variables with *P* value < 0.25 were fitted to the multivariable binary logistic regression model. Finally, variables with *P* value < 0.25 were fitted to the multivariable binary logistic regression model. Finally, variables with

**Results:**

Of the 267 neonates, 48 neonates had perinatal asphyxia, giving a prevalence of 18%. Prolonged labor (AOR = 5.19, 95% CI: 1.73-15.63, *P* value < 0.25 were fitted to the multivariable binary logistic regression model. Finally, variables with *P* value < 0.25 were fitted to the multivariable binary logistic regression model. Finally, variables with *P* value < 0.25 were fitted to the multivariable binary logistic regression model. Finally, variables with *Conclusion and Recommendations*. Prevalence and mortality of asphyxia were high. Prolonged labor, presence of meconium, and preeclampsia were determinant factors for birth asphyxia. Early detection and intervention of high-risk mothers should be carried out by health care providers, and mothers should be monitored with partograph during labor.

## 1. Introduction

Perinatal asphyxia is defined as the inability of the newborn to initiate and sustain enough respiration after delivery and is characterized by a marked impairment of gas exchange [1].

Perinatal asphyxia is caused by a lack of blood flow or gas exchange to the fetus during late pregnancy, during, or after birth as a neonate. When placental (before birth) or pulmonary (immediate after birth) gas exchange is decreased or stopped altogether, there is partial (hypoxia) or complete (anoxia) lack of oxygen to the vital organs. This causes progressive hypoxemia and hypercapnia. If the hypoxemia is severe enough, the tissues and vital organs (muscle, liver, heart, and ultimately the brain) will develop an oxygen debt. Anaerobic glycolysis and lactic acidosis will ensue. Neonatal hypoxic ischemic encephalopathy refers to the neurologic sequelae of perinatal asphyxia [2, 3].

The diagnosis of perinatal asphyxia is made when the umbilical cord arterial pH is <7, the APGAR (Appearance, Pulse, Grimace, Activity, and Respiration) score is 0-3 at the fifth minute, and there are central nervous system manifestations like seizures, lethargy, coma, hypotonia, or hypertonia and multisystem organ dysfunction [4].

The incidence of perinatal asphyxia in developed countries is 2 per 1000 live births, but the rate is 10 times greater in developing countries where there is no adequate access to maternal and neonatal care. Of those asphyxiated neonates, 15-20% will die in the neonatal period and around 25% of survivors will have permanent neurologic deficits [5].

According to the World Health Organization, perinatal asphyxia is one of the three common causes of under-five child mortality (11%) following preterm birth (17%) and pneumonia (15%) [6]. Africa accounts for 11% of the world's total population but with more than 25% of the world's neonatal mortality. Neonatal mortality occurs one in every 4 children in Africa [7]. Because of large neonatal mortalities in Africa, neonatal mortality has remained high globally [8–10]. Neonates born in sub-Saharan Africa have a substantial risk of perinatal asphyxia. Approximately 280,000 neonatal mortalities occur during the first day of life because of birth asphyxia [7, 8, 11, 12].

In Ethiopia, neonatal mortality accounts for 29/1000 live births. Many of these deaths occur during the first 48 hours of age, and still, the reduction in mortality is low [13]. In Ethiopia, perinatal asphyxia is one of the leading causes of neonatal mortality, accounting for 34% [14].

Despite this high mortality and morbidity associated with perinatal asphyxia, the prevalence and associated factors of perinatal asphyxia are not well studied in Ethiopia and there is no report on the prevalence and associated factors of perinatal asphyxia in the study area so far. Therefore, this study is aimed at assessing the prevalence and associated factors of perinatal asphyxia among newborns admitted to Ayder Comprehensive Specialized Hospital in Tigray region.

## 2. Materials and Methods

### 2.1. Study Setting

Ayder Comprehensive Specialized Hospital is found in the Tigray region Mekelle town, which is around 778 km from the capital city Addis-Ababa. It started as a referral and specialized medical center in 2008 GC. It delivers clinical service to more than a population of 8 million in the catchment areas of Tigray, Afar, and southeastern parts of the Amhara regional state. It provides a broad range of medical services to both in- and outpatient for all age groups. It also serves as a teaching hospital to several medical, dental medicine, nursing, midwifery, public health, pharmacy, anaesthesia, and medical laboratory students in both undergraduate and postgraduate programs. It is the second largest hospital in the nation and has more than 500 inpatient beds in the four major departments (internal medicine, pediatrics, gynaecology and obstetrics, and surgery and other specialties). The pediatrics and child health department has 18 specialists (general pediatricians) and six subspecialists. There are 43 residents in the department and 30-40 medical interns rotating every three months.

The NICU (neonatal intensive care unit) ward provides service for approximately 200 neonates per month with a total of 43 beds and one room for KMC (kangaroo mother care). There are 65 BSC nurses, 1 neonatologist, 1 general pediatrician, 4 residents, and 8 interns. It is equipped with 4 radiant warmers, 6 incubators, 5 phototherapy devices, and two mechanical ventilation machines.

### 2.2. Study Design and Period

Institution-based cross-sectional study design was used and data collected to include neonates seen from January 1, 2016, to December 30, 2017.

#### 2.2.1. Source Population

The source population was composed of all neonates admitted to Ayder Comprehensive Specialized Hospital.

#### 2.2.2. Study Population

All neonates admitted to Ayder Comprehensive Specialized Hospital, NICU ward, during the study period and who fulfilled the inclusion criteria were included in the study.

#### 2.2.3. Inclusion Criteria

Inclusion criteria include all newborns admitted to Ayder Comprehensive Specialized Hospital with gestational age ≥ 28 weeks or birth weight ≥ 1000 g.

#### 2.2.4. Exclusion Criteria

Neonates were excluded if they are suffering from major congenital anomalies or syndromes, e.g., NTD (neural tube defect), have incomplete documentation (no maternal or fetal measurement parameters), are kept for observation, and have mothers who took general analgesia.

### 2.3. Sample Size Determination and Sampling Method

The sample size was determined by using a single proportion formula. The sample size determination formula is

(1) $$\begin{array}{c} n=\frac{Z^{2}P\left(1-p\right)}{d^{2}}, \end{array}$$

where *n* is the required sample size, *p* is the proportion, and *d* is the level of precision or acceptable error. 95% confidence interval with a 5% level of precision and 21.1% prevalence of perinatal asphyxia were used in a tertiary hospital in Nigeria [15]. The total sample size was calculated to be 256. With a contingency rate of 10%, the final sample was determined to be 282. Finally, variables with a *P* value < 0.05 were expressed as associated factors of perinatal asphyxia. The study subjects were selected by a systematic sampling method, and relevant information was collected using a checklist.

### 2.4. Study Variables

The dependent and independent variables are provided in Table 1.

### 2.5. Operational Definitions

Neonates are newborn infants who are less than 28 days.

Perinatal asphyxia is the inability of the newborn to initiate and sustain adequate respiration after delivery.

APGAR score is a score used as a part of early assessment of a newborn.

Perinatal asphyxia is considered when the 5^th^ APGAR score is <7 or a neonate did not cry or needed resuscitation.

HIE (hypoxic ischemic encephalopathy) is a central nervous system dysfunction during the neonatal period, and it is due to ischemic and hypoxic insult.

Prolonged labor is the total duration of labor > 24 hours.

Congenital malformation is a physical defect present in a newborn at birth that results in central nervous system depression.

### 2.6. Data Collection Procedures

Prestructured data collection format was used to collect the information. Data was collected by medical interns. Relevant information was obtained which includes neonatal information (gender, gestational age, birth weight, and APGAR score), maternal information (age, parity of mothers, residence, place of delivery, mode of delivery, and problems during pregnancy or labor).

### 2.7. Data Processing and Analysis

Data were entered into SPSS (Statistical Package for the Social Sciences) version 20 (Armonk, NY: IBM Corp), cleaned, coded, and checked for normality and completeness before analysis. Descriptive statistics was used to determine the prevalence of birth asphyxia and sociodemographic as well as obstetrics history. Bivariate and multiple binary logistic regression analysis was carried out to identify the associated factors of PNA. Variables with a *P* value < 0.25 during bivariate analysis were included to multivariable logistic regression model. Finally, variables with a *P* value < 0.05 were expressed as associated factors for perinatal asphyxia.

### 2.8. Ethical Considerations

Ethical clearance was obtained from the Institutional Review Board (IRB) of the College of Health Sciences of Mekelle University. Permission was taken from Ayder Comprehensive Specialized Hospital medical director offices; a support letter from the chief clinical director was obtained.

## 3. Results

A total of 3403 neonates were admitted to the NICU during the study period, and a total of 282 neonate medical records were collected using a standardized random sampling approach and 267 (94.7%) neonate medical records had complete data, and 15 patients were omitted due to incomplete data. Of the 267 neonates, 48 had perinatal asphyxia, giving us a prevalence of 18%. Majority of the study neonates' mothers (62.2%, *n* = 166) were aged 20-35. More than half of the mothers (57.6%, *n* = 154) were from urban area. Of the study neonates, 58.8% (*n* = 157) were males. Most of the neonates (82.2%, *n* = 221) were admitted in the first 72 hours of age. The mean age of the study neonates at admission was 2.47 days with SD (±3 days) and 1.25 (±0.7 days) for those neonates with PNA. The majority of the mothers of PNA neonates (28, 58.3%) were between 20 and 35 years of age. Almost all neonates (98%, *n* = 47) with PNA were admitted within the first 72 hours of age (Table 1).

### 3.1. Clinical Characteristics of Study Participants

Majority of the mothers (60.3%, *n* = 161) were multiparous. Almost all mothers (98.5%, *n* = 263) had ANC follow-up, and 93.6% (*n* = 250) had normal duration of labor. Of all mothers, 95.9% (*n* = 256) had intrapartum membrane rupture and 6.4% (*n* = 17) of them had preeclampsia. With respect to amniotic fluid status, twenty-two (8.2%) had MSAF. Majority (61.4%, *n* = 164) had normal vaginal delivery. Although 5.6% (*n* = 15) neonates were born with birth weight greater than or equal to 4000 g, 59.2% (*n* = 158) were weighing 2500-3999 g.

Among the mothers of neonates with PNA, 52.1% (*n* = 25) were from rural area, while 58.3% (*n* = 28) were multiparous. Of all these neonates, 58.3% (*n* = 28) were born in tertiary hospitals. A significant number of neonates (8, 16.6%) had MSAF. Most of the mothers (83.3%, *n* = 40) had normal labor and delivery history, and 62.5% (*n* = 30) of them gave birth via SVD. Majority of the asphyxiated neonates (58.3%, *n* = 28) were males (Table 2).

### 3.2. Clinical Features and Outcomes of Asphyxiated Neonates

Thirty-three neonates (68.75%) had a score of 4–6 at the 5^th^ minute, and 27.15% (*n* = 13) had no record of APGAR score but needed resuscitation (did not cry). More than half (26, 54.17%) were admitted with stage II PNA (Table 3). When we see the outcome of asphyxiated neonates, 56.25% (28) discharged improved, while 37.5% (*n* = 18) newborns died, resulting in a case fatality rate of 37.5%. From the asphyxiated neonates who died, 61% (*n* = 11) were stage III, one-third of them (33.3%, *n* = 26) were stage II, and one was stage I PNA. The mean hospital stay of PNA patients was 9.42 days with SD (±8.81).

Nine PNA neonates (18.8%) developed seizure. Of those neonates who had seizure, 66.7% (*n* = 6) were stage III and 33.3% (*n* = 3) were stage II.

### 3.3. Bivariate and Multivariable Analysis

In bivariate regression analysis, residence, gestational age, preeclampsia, place of delivery, birth weight, presence of meconium, and duration of labor were significantly associated factors of PNA (Table 4). But on multivariable logistic regression analysis, preeclampsia, presence of meconium, and duration of labor were statistically significant associated factors of PNA. Neonates born to mothers who had preeclampsia were 7.94 times more likely to develop PNA as compared to neonates from mothers without preeclampsia (AOR = 7.94, 95% CI: 2.22-28.37).

Neonates born to mothers with prolonged duration of labor were 5 times more likely to have PNA (AOR = 5.19, 95% CI: 1.73-15.63).

Neonates who were born with meconium-stained amniotic fluid were 4 times more likely to have perinatal asphyxia as compared to those neonates delivered with clear amniotic fluid (AOR = 4.17, 95% CI: 1.34-12.98).

## 4. Discussion

In the present study, the prevalence of perinatal asphyxia was found to be 18%, which is higher than in developed countries, the latter of which has reduced it to less than 0.1% [16]. However, similarly high prevalence rate was seen in general hospitals of Tigray, 22.1% [17]. Moreover, it is comparable with the studies done in other African countries like Gusau, Nigeria (21.1%) [15], and Dar es Salaam, Tanzania (21.1%) [18]. But it was lower than the study conducted in Dilla, Southern Ethiopia, which was 32.8% [19]. This high rate of variation could be due to differences in the methodology, the use of different definitions of birth asphyxia in different settings, the difference in the study facilities, and maybe due to resource disparity or the study participants' economic status. It could also be attributed to the difference in the availability of skilled workers.

In this study, more males than females were affected by perinatal asphyxia (58.3%). This is consistent with the report from Bangladesh (60.8%) [20], Dow University of Health Sciences, Karachi (61.3%) [21], and 60.3% in Nigeria [22]. This could be explained by the protective effect of the additional “x” chromosome [23].

The case fatality rate of perinatal asphyxia was 37.5%. This is comparable with the study conducted in Sri Lanka which was 40.6% [24]. But it is higher than the reports from Enugu, southeast Nigeria (18%) [25], Gusau, Nigeria (25.5%) [15], and Birnin Kudu, Nigeria (10.3%) [26]. However, the frequency found in this study is lower than the 62.5% fatality rate from Dar es Salaam, Tanzania [18].

The disparity could be attributable to the difference in hospital setup (some may be better equipped) and the difference in health care provider skills and birth asphyxia severity. Although this study has reported high fatality rate, perinatal asphyxia has contributed to a low overall neonatal mortality rate (6.7%, *N* = 18) which is lower than what has been reported in other studies.

Most of the neonates with stage III perinatal asphyxia (84.6%) died. This is higher than the reported 66.7% from Enugu, southeast Nigeria [25], and 44.4% from Karnataka, India [27]. The reason could be due to the difference in the health facility setup and the presence of multiorgan failure associated with hypoxic-ischemic encephalopathy.

The current study revealed that the odds of developing perinatal asphyxia was 4 times higher in neonates of mothers who had meconium-stained amniotic fluid than those without meconium-stained amniotic fluid. Such was demonstrated in the general hospitals of Tigray [17] and also [20, 28, 29]. MASF is often associated with fetal hypoxia which promotes meconium discharge in amniotic fluid, gasping and aspiration of MSAF, and also changes in the vascular muscular of pulmonary blood vessels of the fetus [30].

The odds of developing perinatal asphyxia was 5.19 times higher in a newborn whose mother had prolonged duration of labor. This is consistent with reports from different hospitals in Ethiopia including general hospitals in Tigray, Dessie, and Dire Dawa [17, 31, 32] and in other African countries like Cameroon [33] and Bayero University Kano, Nigeria [22]. The reason could be delayed labor which might be causing the fetus to be involved in labor for a long time that carries a higher risk of birth trauma and asphyxia [34]. Strict follow-up of laboring mother with partograph may ameliorate the situation.

Neonates born to mothers who had preeclampsia were 7.94 times more likely to have perinatal asphyxia than neonates from mothers without preeclampsia. This is in agreement with studies done in universities in Nigeria [22], Nairobi [35], and Pakistan [36]. The reason why preeclampsia is a risk factor for perinatal asphyxia could be due to decreased blood flow to the fetus that may lead to hypoxia and finally perinatal asphyxia.

As this study has shown high prevalence and also alarmingly significant mortality in neonates with PNA, good obstetric interventions and proper care of neonates in the neonatal intensive care unit are mandatory.

### 4.1. Strength of the Study

This is the first PNA study in our hospital, and it was able to show the prevalence, associated factors, and outcome of asphyxiated neonates. The study was designed with random sampling technique. Moreover, neonates were included from both rural and urban areas of residence.

### 4.2. Limitations of the Study

This study had some important limitations because it was conducted in a tertiary care hospital where significant numbers of patients were referred being critical; therefore, this prevalence may not reflect the overall prevalence of the community.

Furthermore, this study does not show cause-and-effect relationships because of the cross-sectional study design.

Measurement of fetal or neonatal arterial blood gas would give a stricter and more precise definition of PNA but our hospital's setup could not give such services because of resource constraints.

## 5. Conclusion

In conclusion, the prevalence of perinatal asphyxia was high. The case fatality rate of perinatal asphyxia was alarmingly high. Prolonged labor, presence of meconium-stained amniotic fluid, and preeclampsia were predictors of perinatal asphyxia. Early detection and intervention of high-risk mothers should be carried out by health care providers, and mothers should be monitored with partograph during labor.

## Figures and Tables

**Table 1:** Study variables.

Dependent	Independent
Perinatal asphyxia	Age of the mother
	Residence
	Age of neonates at admission
	Sex of newborn
	Gestational age in weeks
	Birth weight in grams
	Parity
	ANC follow-up (antenatal care follow-up)
	Birth history
	Comorbidities (diabetes, preeclampsia)
	Type of labor (spontaneous or induced)
	Duration of labor
	Place of delivery
	PROM (premature rupture of membranes)
	MSAF (meconium-stained amniotic fluid)
	Fetal presentation
	Mode of delivery
	APGAR score (Appearance, Pulse, Grimace, Activity, and Respiration)
	Duration of hospital stay

**Table 2:** Sociodemographic and clinical characteristics of mothers and neonates at ACSH, Ethiopia, January 1, 2016–December 30, 2017 (*n* = 267).

Variables	Perinatal asphyxia		
	Yes (48), *n* (%)	No (219), *n* (%)	*n* (%)
Age of mothers			
<20	2 (4.2)	11 (5)	13 (4.8)
20-35	28 (58.3)	138 (63)	166 (62.2)
>35	18 (37.5)	70 (32)	88 (33)
Residence of mothers			
Urban	23 (47.9)	131 (59.8)	154 (58.8)
Rural	25 (52.1)	88 (40.1)	113 (42.2)
Sex of neonates			
Male	28 (58.3)	129 (58.9)	157 (58.8)
Female	20 (41.7)	90 (41.1)	110 (41.2)
Age of neonate at admission			
0-72 h	47 (98)	174 (79.5)	221 (82.2)
3-7 days	1 (2)	20 (9.1)	21 (7.8)
>7 days	0 (0.0)	25 (11.4)	25 (10)
Weight			
≥4000	2 (4.16)	13 (5.9)	15 (4.5)
2500-3999	37 (77.1)	121 (55.3)	158 (59.2)
1500-2499	10 (20.8)	72 (32.9)	82 (30.7)
1000-1499	2 (4.2)	10 (4.6)	12 (5.6)
Parity			
Nulliparous	20 (41.7)	86 (39.3)	106 (39.7)
Multipara	28 (58.3)	133 (60.7)	161 (60.3)
ANC follow-up			
Yes	48 (100)	215 (98.2)	263 (98.5)
No	0 (0.0)	4 (1.8)	4 (1.5)
Place of delivery			
Tertiary hospital	28 (58.3)	129 (58.9)	157 (58.8)
Primary and general hospitals	6 (12.5)	41 (18.7)	47 (17.6)
Health center	13 (27.1)	44 (16.4)	57 (21.3)
Home delivery	1 (2.1)	5 (2.3)	6 (2.2)
Previous birth history			
Abortion	2 (4.2)	18 (8.2)	20 (7.5)
Still birth	1 (2.1)	7 (3.2)	8 (3)
Neonatal death	1 (2.1)	4 (1.8)	5 (1.9)
None	44 (91.7)	190 (86.8)	234 (87.6)
Comorbidity			
Preeclampsia	7 (14.6)	10 (4.6)	17 (6.4)
DM	0 (0.0)	6 (2.72)	6 (2.2)
None	41 (87.4)	202 (92.2)	244 (91.4)
Type of labor			
Spontaneous	42 (87.5)	193 (88.1)	235 (88)
Induced	6 (12.5)	24 (11.0)	30 (11.2)
Augmented	0	2 (0.9)	2 (0.8)
Duration of labor			
Normal	40 (83.3)	210 (95.9)	251 (94)
Prolonged	8 (16.6)	9 (4.1)	17 (6.4)
PROM			
Yes	1 (2.1)	9 (4.1)	11 (4.1)
No	46 (97.9)	210 (95.9)	256 (95.9)
MSAF			
Yes	8 (16.6)	14 (6.4)	22 (8.2)
No	40 (83.3)	205 (93.6)	245 (91.8)
Fetal presentation			
Cephalic	46 (95.8)	212 (96.8)	258 (96.6)
Noncephalic	2 (4.2)	7 (3.2)	9 (3.4)
Mode of delivery			
C/S	16 (33.3)	78 (35.6)	94 (35.2)
SVD	30 (62.5)	134 (61.2)	164 (61.4)
Instrumental	2 (4.2)	7 (3.2)	9 (3.4)
Gestational age			
Preterm	9 (18.8)	67 (30.6)	76 (28.4)
Term	38 (79.2)	141 (64.4)	179 (67)
Postterm	1 (2.1)	11 (5.0)	12 (4.6)

Abbreviations: PROM: premature rupture of membrane; MSAF: meconium-stained amniotic fluid; ANC: antenatal care.

**Table 3:** Clinical status of asphyxiated neonates, January 01, 2016–December 30, 2017 (*n* = 48).

Clinical factors	Frequency	%
Perinatal asphyxia		
Yes	48	18.0
No	219	82.0
SARNAT stage of PNA		
Stage I	9	18.75
Stage II	26	54.17
Stage III	13	27.1
APGAR score at the 5^th^ minute		
0–3	2	4.2
4–6	33	68.75
Needed resuscitation (did not cry)	13	27.1

Abbreviation: PNA: perinatal asphyxia.

**Table 4:** Bivariate and multivariable logistic regression model showing predictors of birth asphyxia among babies admitted in ACSH, January 1, 2016–December 30, 2017.

Characteristics	PNA	Non-PNA	At 95% CI	At 95% CI	*P* value
			COR	AOR	
Preeclampsia					
No	41	209	Ref.	Ref.	
Yes	7	10	3.57 (1.28-9.91)	7.94 (2.22-28.37)	0.001
Residence					
Urban	23	131	Ref.	Ref.	
Rural	25	88	1.79 (1.150-0.98-3.25)	1.7 (0.87-3.37)	0.114
Preterm	9	67	Ref.	Ref.	
Term	38	141	0.50 (0.23-1.09)	0.67 (0.22-2.02)	0.486
Postterm	1	11	1.48 (0.17-12.84)	2.87 (0.26-32.47)	0.393
Place of delivery					
Tertiary hospital	28	129	Ref.		
Primary and general hospitals (district)	6	41	1.13 (0.46-2.8)	0.84 (0.29-2.4)	0.75
Health center	13	44	0.61 (0.29-1.27)	0.58 (0.23-1.47)	0.255
Home	1	5	0.99 (0.11-8.85)	1.02 (0.1-10.34)	0.98
Birth weight					
2500-399 g	34	124	Ref.	Ref.	
1500-2499 g	10	72	1.97 (0.92-4.23)	1.59 (0.57-4.41)	0.372
1000-1499 g	2	10	1.37 (0.28-6.56)	2.65 (0.28-25.01)	0.395
≥4000 g	2	13	1.78 (0.38-8.28)	0.56 (0.18-5.12)	0.96
MSAF					
Yes	8	14	2.92 (1.15-7.44)	4.17 (1.34-12.98)	0.014
No	40	205	Ref.	Ref.	
Duration of labor					
Normal	40	210	Ref.	Ref.	
Prolonged	8	9	4.7 (1.7-12.82)	5.19 (1.73-15.63)	0.003

Abbreviation: MSAF: meconium-stained amniotic fluid.

## Data Availability

All important data are included in the manuscript.
